# Advances in epigenetic studies of plant cadmium stress

**DOI:** 10.3389/fpls.2024.1489155

**Published:** 2025-05-30

**Authors:** Yonggang Gao, TZufeng Wang, Cheng Zhao

**Affiliations:** ^1^ Guangdong Laboratory of Lingnan Modern Agriculture, Key Laboratory of Synthetic Biology, Ministry of Agriculture and Rural Affairs, Agricultural Genomics Institute, Chinese Academy of Agricultural Sciences, Shenzhen, China; ^2^ Institute of Agricultural Genomics, Center for Synthetic, Biology, Chinese Academy of Agricultural Sciences Agricultural Genomes Institute, Shenzhen, China; ^3^ College of Agriculture and Natural Resources, National Chung Hsing University, Taizhong, Taiwan, China

**Keywords:** heavy metal, cadmium, epigenetics, DNA methylation, plant

## Abstract

As the global population continues to grow, achieving ecological sustainability and ensuring food production have become urgent challenges. Among various environmental stresses, heavy metals, particularly cadmium (Cd), pose a significant threat to plant growth and development. Breeding cadmium-resistant crop varieties that minimize Cd accumulation is therefore crucial for promoting sustainable agriculture. In response to Cd stress, plants undergo a series of regulatory mechanisms, including DNA methylation, chromatin remodeling, and histone acetylation, to mitigate cellular damage. Understanding the epigenetic responses of plants to cadmium stress is a key research area that holds substantial significance for both agriculture and environmental biology. This article reviews the current research on plant responses to cadmium stress and the underlying mechanisms of their epigenetic responses, aiming to provide theoretical insights for analyzing the epigenetic mechanisms of heavy metal stress in major crops. We can leverage genomics, single-cell sequencing, stereo-seq, and other advanced technologies in conjunction with epigenomics, plant genetics and molecular biology techniques to conduct comprehensive and in-depth studies on the epigenetic changes that occur in plants following Cd exposure. Systematically elucidating the molecular mechanisms by which plants perceive and respond to Cd stress will aid in the development of more effective bioremediation strategies for heavy metal-contaminated soils and facilitate.

## 1 Introduction

Heavy metal soil pollution has significantly increased in certain regions due to the rapid development of domestic industrialization (Holubek et al., 2020). The excessive accumulation of cadmium can harm crop growth and development, resulting in reduced yield and quality. This poses a threat to human health through the transmission of food chains (Holubek et al., 2020). Cadmium is a non-essential heavy metal that exhibits high toxicity to plants. Over the course of long-term evolution, plants have developed a complex array of mechanisms to mitigate cadmium stress (Qi et al., 2018). Research on plant response to cadmium stress is making continuous progress, with successive identification of genes related to this response, thereby enhancing our understanding of how plants cope with cadmium stress.

Plants have evolved specific mechanisms for perceiving, transporting, and detoxifying cadmium (Zhang et al., 2012; Jiang et al., 2020). Cd is a limiting factor for crop growth because it reduces the chlorophyll content in plants, which negatively impacts normal photosynthesis (Clemens and Ma, 2016). Additionally, it induces the production of high levels of reactive oxygen species, resulting in premature aging of plants through peroxidation (Wang et al., 2015; Wu et al., 2021). The extent of Cd toxicity to plants depends on several factors, such as the plant species, Cd concentration, and duration of Cd stress. There is a delicate balance of nutrient elements within plants, which is disrupted by excessive Cd. When Cd enters the plant, it competes for binding sites with other essential nutrient elements, leading to changes in the internal element concentrations of plants. Specifically, Cd interacts with certain divalent metals such as Ca^2+^, Fe^2+^, Mn^2+^, and Zn^2+^, affecting the absorption of these beneficial elements (Wang et al., 2015; Zhang et al., 2022).Therefore, the need to study the effects of Cd is becoming more and more urgent.

Abiotic stresses include variations in temperature, drought, salinity, nutrition, heavy metal toxicity etc (Luo and He, 2018). Epigenetic regulation is essential for plant processes such as growth, development, reproduction, as well as for enhancing adaptability to environmental stresses such as drought, temperature, salinity and heavy metal stress (Li et al., 2021; Duarte-Aké et al., 2023; Mukhtar et al., 2024). The impact of these epigenetic mechanisms is directly reflected in crop productivity, yield and quality. Numerous studies have been conducted both domestically and internationally to investigate various aspects of plant responses to cadmium, such as uptake, accumulation, transport, the toxic effects, as well as the physiological and molecular mechanisms underlying cadmium resistance (Reinders et al., 2005; Kazemi et al., 2020; Zhang et al., 2022). Aina et al. (2004) carried out a comparative analysis of DNA methylation levels between clover (*Trifolium repens* L.), a plant sensitive to chromium (Cr), nickel (Ni) and cadmium (Cd), and hemp (*Cannabis sativa* L.), which exhibits partial tolerance to these heavy metals (HMs). Their research showed that in the absence of HM stress, hemp roots had a significantly higher methylation level than clover roots. Similarly, Gullì et al. (2018) observed that Noccaea caerulescens plants, a known nickel hyperaccumulator species, displayed significant hypermethylation at the genomic level when grown under high Ni concentrations, in contrast to Arabidopsis thaliana plants, which are sensitive to Ni and showed less methylation under similar conditions. Furthermore, Gullì et al. (2018) showed that the genes MET1, DRM2 and HDA8, which play a crucial role in DNA methylation and histone modification, showed different expression patterns between *N. caerulescens* and *A. thaliana*.

However, relatively little research has focused on the epigenetic regulatory mechanisms involved in plant responses to cadmium stress and the primary metabolic processes they trigger. Cadmium pollution is an urgent and significant environmental problem. Undertaking physiological and molecular research on how plants respond to cadmium stress and elucidating the underlying mechanisms may help in the development of crops with reduced ability to absorb and accumulate cadmium. This article presents a comprehensive examination of the epigenetic alterations and response mechanisms of plants subjected to cadmium-induced stress. It furnishes a theoretical foundation for comprehending the metabolic disparities in plants under cadmium stress, provides insights into plant resistance strategies against cadmium, and serves as guidance for future endeavors in crop breeding and environmental remediation.

## 2 The present state of research on the genetic mechanisms of plant cadmium tolerance

In recent years, numerous studies have been conducted to elucidate the genetic mechanisms that underlie plant cadmium tolerance. Cadmium, a toxic heavy metal, poses significant threats to both human health and the environment (Muhammad et al., 2016). Therefore, comprehending plants’ ability to tolerate and adapt to cadmium stress is crucial for developing strategies in phytoremediation and crop improvement. Throughout the years, research endeavors have concentrated on the identification of pivotal genetic factors and pathways implicated in plant cadmium tolerance (Shi et al., 2022). Molecular techniques have identified numerous genes and proteins associated with cadmium tolerance. These genes play critical roles in regulating diverse physiological processes and detoxification mechanisms in plants (Lu et al., 2021). Further investigations are required to comprehensively elucidate the intricate regulatory networks associated with plant cadmium tolerance. Understanding the genetic mechanisms of plant cadmium tolerance will provide valuable insights for developing strategies to enhance cadmium tolerance (Hejátko et al., 2017; Arribas et al., 2018; Wu et al., 2020).

Developing new varieties with low cadmium levels is an effective and eco-friendly approach to mitigate the risk of cadmium pollution in plants. To accomplish this objective, it is essential to have a thorough understanding of both the mechanisms by which plants respond to cadmium stress and the regulation of cadmium absorption, transport, sequestration, and other vital processes.

Plants have evolved intricate mechanisms to adapt to cadmium stress through long-term evolution (Liang et al., 2020). Ongoing research is continuously uncovering genes associated with plant response to cadmium stress, thereby enhancing our comprehension of plant responses to this stress (Edelheit et al., 2019). Substantial advancements have been made in recent years in the elucidation of the physiological and molecular mechanisms underlying cadmium transport and tolerance in plants. By considering the correlation between metal concentrations in soil and plants, plants can be classified into three categories: excluders, indicators, and hyperaccumulators (Anderson et al., 2018; Shao et al., 2021).

In light of rapid advancements in molecular biology and biotechnology, researchers have been undertaking fundamental studies to deepen our understanding of plant responses to cadmium stress and the interactions between cadmium and plants (Du et al., 2020). The transportation and distribution of cadmium in different plant organs, such as roots, stems, leaves, and fruits, will be discussed (Lu et al., 2021). The coordinated response of multiple genes in plants to alleviate cadmium stress will be explored. The epigenetic mechanisms contributing to plant cadmium tolerance will be examined. Extensive and intensive research has been conducted on the transport and accumulation of cadmium in model plants such as Arabidopsis and rice (Yuan et al., 2020). This includes investigating processes such as root uptake, translocation from the root stele to the shoot, redistribution through the stem and nodes, and subsequent enrichment and translocation to grains via the stem’s vascular bundles (Li et al., 2023).

**Figure 1:**
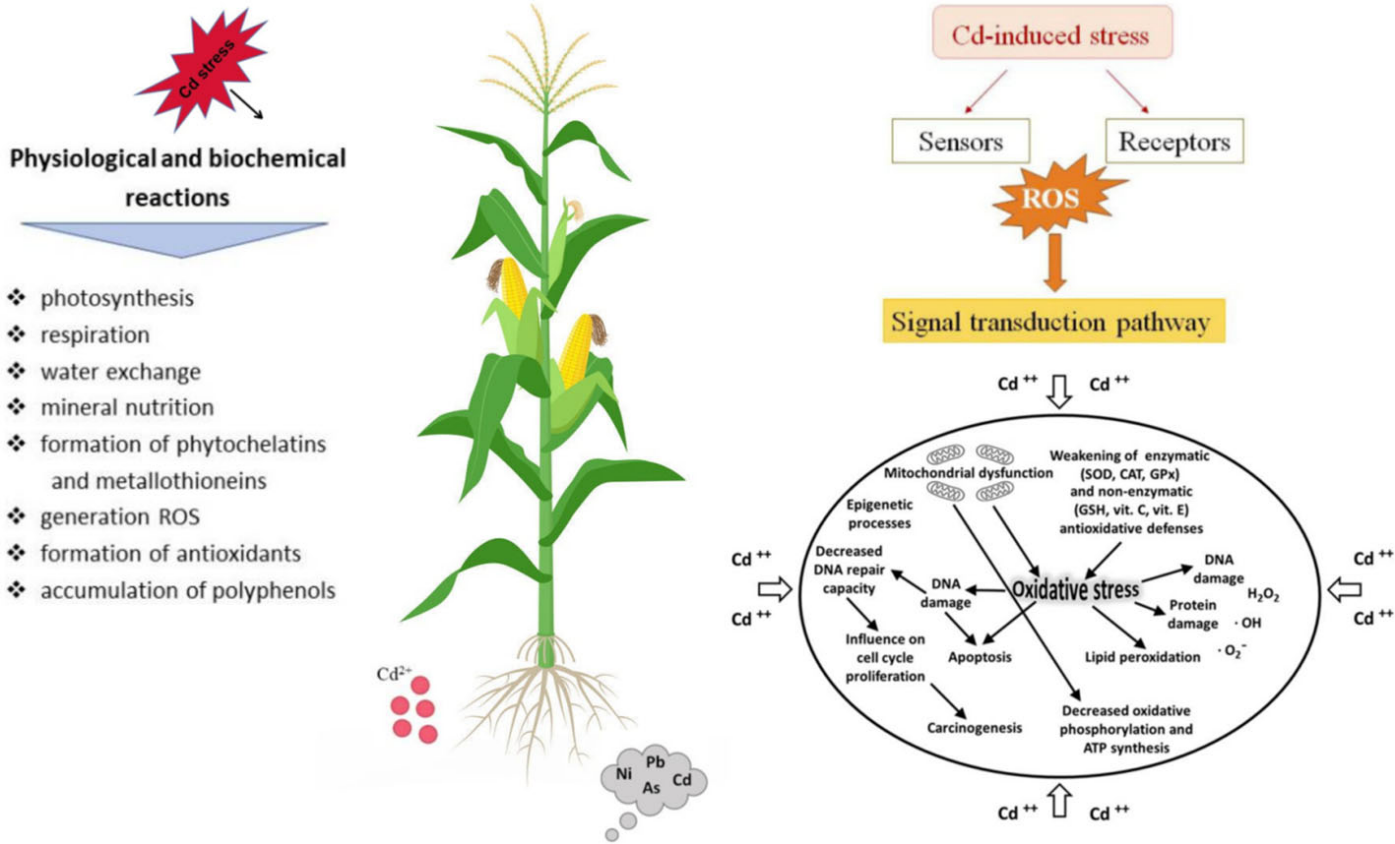
Physiological responses and molecular signaling patterns of plants under cadmium (Cd) stress.

The stress signal is transmitted within the cell to initiate a response. Cadmium ionic can interact with various cellular components and disrupt normal function (Wu et al., 2022). Superoxide dismutase (SOD), catalase (CAT), and glutathione peroxidase (GPx) are enzymes that typically protect the cell from ROS but may be compromised under Cd stress (Goncharuk et al., 2023). Cd stress can cause oxidative damage to cells. Cd can cause DNA damage, which may lead to mutations or cell death if not repaired. Cellular responses to Cd stress, such as changes in photosynthesis, respiration, and water exchange (**Figure 1**).

The mechanism by which plants sense cadmium and its associated signaling transduction pathways. The cellular and molecular mechanisms underlying the toxic effects of cadmium on plants (Yuan et al., 2020). Numerous metal transport proteins involved in cadmium uptake, transport, and detoxification have been cloned in rice and Arabidopsis. These proteins include iron (Fe)-regulated transporters such as *OsIRT1* and *OsIRT2*, natural resistance-associated macrophage protein (*OsNRAMP1*), at *NRAMP1*, *AtNRAMP3*, and *AtNRAMP4*, zinc (Zn)/iron-regulated transporters such as *OsZIP1* and *OsZIP3*, as well as *CAL1*, *osNRAMP5*, *HvNRAMP5*, cation/calcium (Ca) exchanger (*OsCCX2*), heavy metal ATPases (*OsHMA2*) and *OsHMA3*, low-affinity cation transporter (*OsLCT1*), and oligopeptide transporter *OPT3*. The identification of these genes has laid an important theoretical groundwork for the molecular breeding of crops with low cadmium accumulation (Zheng et al., 2021; Sun et al., 2021).

Currently, research has focused on plant responses to cadmium stress in two main areas. Building upon the identified cadmium stress-responsive genes, biotechnological approaches can be utilized to develop crop materials that accumulate high levels of cadmium and exhibit tolerance to cadmium (Holubek et al., 2020). This requires the establishment of a transformation system for cadmium-enriched plants, which can subsequently be employed for remediating cadmium-contaminated soils in various locations, including mines, smelting sites, and areas involved in cadmium product manufacturing (Fang et al., 2013; Wang et al., 2018; Repkina et al., 2019). By screening for crop varieties with low cadmium accumulation, developing relevant molecular markers, and combining multiple genes associated with low cadmium accumulation through molecular breeding, it is possible to create crop varieties that exhibit low cadmium accumulation, high yield, and excellent quality.

Recently, researchers have investigated the capabilities of sunflowers, Indian mustard, and red gum in removing cadmium from contaminated soils and water. Furthermore, the efficient utilization of nano TiO_2_ and Al_2_O_3_ for cadmium removal from both wastewater and soil has been demonstrated (Trela-Makowej et al., 2023). Previous research has demonstrated that plants react to Cd pollution by experiencing various stress responses, including oxidative stress, changes in enzyme activity, and disruptions in plant signaling substances like hormones and calcium ions (Cataldo et al., 1981; Zhang et al., 2006; Tkalec et al., 2014; Cheng et al., 2021). These responses damage the photosynthetic system, induce lipid peroxidation and cell injuries, alter enzyme activity, induce endoplasmic reticulum stress, cause protein degradation, DNA damage, and mutations, subsequently affecting physiological and biochemical metabolic processes. Ultimately, these factors inhibit plant growth and may even lead to plant death (Zhang et al., 2015; Frye et al., 2018; Zhao et al., 2020; Quadros et al., 2022). Plants have developed an intricate suite of genes that are pivotal in the processes of cadmium (Cd) uptake, transport, sequestration, and detoxification (Qin et al., 2017; Song et al., 2021). These genes constitute a sophisticated network of mechanisms essential for plants to endure Cd-induced stress, especially in environments where contamination is prevalent (**Table 1**). Gaining a comprehensive understanding of the functions and regulatory mechanisms of these genes is vital for the development of plant varieties with heightened Cd tolerance and for the deployment of efficacious phytoremediation strategies. Advances in genetic engineering and selective breeding can capitalize on this knowledge to bolster plants' resistance to Cd toxicity, thereby tapping into their capacity for environmental remediation (Wang et al., 2022; Shilpa et al., 2024).

**Table 1:** Genes involved in Cd uptake, transport, sequestration, and detoxification in plants.

Category	Gene Name	Main Expression Organ	Function	Reference
Arabidopsis Arabidopsis thaliana	*AtNRAMP 1*	Root, leaf	Cd uptake	Thomine et al., 2000.
	*AtNRAMP 3*	Root, leaf	Cd uptake	Thomine et al., 2000.
	*AtNRAMP 4*	Root, leaf	Cd uptake	Thomine et al., 2000.
	*AtHMA1*	Root, Stem, leaf	Cd root-to-shoot translocation	Kim et al., 2009.
	*AtHMA2*	Root, shoot	Cd sequestrating in vacuoles	Zuo et al., 2023
	*AtHMA3*	Root, Stem, leaf	Cd and Zn root-to-shoot translocation	Zuo et al., 2023.
	*AtHMA4*	Root, shoot	Cd and Zn root-to-shoot translocation	Rebecca et al., 2005
	*AtPDR8*	Root, leaf	Cd efflux	Kim et al., 2007.
	*PvHMA2.1*	Root, Stem, leaf	Cd uptake	Zang et al., 2023.
Rice	*OsZIP1*	Root	Cd and Zn transport	Liu X. S. et al., 2019.
	*OsZIP3*	Stem	Cd accumulation	Zhang et al., 2022.
	*OsZIP6*	Root, stem	Cd transport	Kavitha et al., 2015.
	*OsZIP7*	Root, node	Cd and Zn accumulation	Tan et al., 2019.
	*OsZIP9*	Root	Cd and Zn uptake	Tan et al., 2020.
	*OsNRAMP1*	Root, leaf	Cd absorption by root	Chang et al., 2020.
	*OsNRAMP2*	Shoot	Cd transport and accumulation	Zhao et al., 2018.
	*OsThi9*	Root, Stem, leaf	Cd accumulation	Liu et al., 2022.
	*OsHIPP9*	Root, leaf	Cd transport	Xiong et al., 2023.
Wheat	*TaTPR2*	Root, Stem	Cd absorption by root	Wang et al., 2024.
	*TpIRT1*	Root, Stem, leaf	Cd and Zn uptake	Jiang et al., 2021.
	*TaHMA2*	Root leaf	Cd root-to-shoot translocation	Xiang et al., 2015.
	*TaWRKY22- TaCOPT3D*	Root Stem, leaf	Cd and Zn transport	Liu et al., 2022.
	*AetSRG1*	Root Stem, leaf	long-distance Cd translocation	Wei J. et al., 2022.
	*WRKY74*	Root, Stem, leaf	Increase Cd translocation from roots to shoots	Li et al., 2022.
Maize	*ZmHMA3*	Root, leaf	Cd and Zn accumulation	Tang et al., 2021.
	*ZmWRKY4*	Root, leaf	Cd and Zn uptake	Hong et al., 2017.
	*ZmWRKY64*	Root, leaf	Cd absorption, transport	Gu et al., 2024.
	*ZmOXS2b*	Root, Stem, leaf	Cd absorption by root	He et al., 2016.
	*ZmO2L1*	Root, shoot	Cd transport and accumulation	He et al., 2016.
	*ZmbHLH105*	Root	Cd transport and accumulation	Meng et al., 2024.
	*ZmHMT3*	Root, Stem	Cd accumulation	Lin et al., 2024.
Soybean	*GmWRKY142*	Root, Stem, leaf	Cd and Zn uptake	Cai et al., 2020.
	*GmWRKY172*	roots, leaves, flowers, shoots and seeds	Cd translocation from roots to shoots and seeds	Xian et al., 2023.
	*GmHMA1*	Root, Stem, leaf	Cd and Zn root-to-shoot translocation	Eduardo et al., 2012.
	*GmHMA3*	Root, leaf	Cd and Zn root-to-shoot translocation	Wang et al., 2018.
	*GmNHX1*	Stem	Cd and Zn transport	Yang et al., 2017.
	*GmIRT1.1*	Root	Fe, Mn and Cd transport	Gong et al., 2024.

## 3 Epigenetic regulation of gene expression in plants under cadmium stress

Epigenetics refers to changes in gene expression that do not result from alterations in the DNA nucleotide sequence (Tiwari and Lata, 2018).The primary mechanisms involved in the regulation of gene expression through epigenetics include DNA methylation, post-translational modifications of histones, small non-coding RNA molecules (such as microRNAs, miRNAs) that can disrupt gene transcription and/or translation, and the organization of DNA around nucleosomes (Qiu et al., 2008; Kim et al., 2009; Jozefczak et al., 2012; Shen et al., 2019; Deng et al., 2022). Enzymes involved in the processes of epigenetics include DNA methyltransferases, histone methyltransferases, histone acetyltransferases, and histone deacetylases (Sarah et al., 2022).

Cadmium, a non-essential heavy metal, has a high toxicity towards plants. Plants have developed specialized mechanisms for sensing, transporting, and detoxifying cadmium (Clemens and Ma, 2016; Mao et al., 2018; Poonam et al., 2020). Recent research has identified numerous transport proteins involved in cadmium uptake, transport, and detoxification (Qiao et al., 2015; Trela-Makowej et al., 2024). However, the complex transcriptional regulatory network underlying cadmium response is still not fully understood. Increasing evidence suggests that epigenetic regulation, involving DNA methylation, lncRNA, miRNA, and kinases, plays a significant role in Cd-induced transcriptional responses and contributes to cadmium tolerance (Liu et al., 2019; Sarah et al., 2022; Han et al., 2023). Studying these signal transduction and response mechanisms at both the transcriptional and post-transcriptional levels will improve our understanding of regulatory pathways and provide a foundation for developing effective strategies to reduce plant cadmium accumulation (Ojolo et al., 2018; Shim et al., 2020; (He et al., 2021; Liu et al., 2023).

Understanding the processes and pathways involved in plant cadmium accumulation and response requires the identification of target genes regulated by epigenetic mechanisms and RNA regulation. Extensive research has currently been conducted on cadmium accumulation and response in rice and Arabidopsis (Nie, 2021; Shi et al., 2022). However, different plant species may have many unknown components and mechanisms that play significant roles in cadmium accumulation and tolerance. For instance, certain plants with the ability to hyper accumulate cadmium serve as valuable research materials for discovering new mechanisms of cadmium accumulation, isolation, and detoxification from heavily contaminated soils (Liu et al., 2021). These findings could potentially provide valuable insights into novel mechanisms for the accumulation and detoxification of high levels of cadmium in plants.

### 3.1 The role of DNA methylation in cadmium stress

DNA methylation refers to the process where a methyl group is covalently bonded to the 5-methylcytosine of DNA. This reaction is catalyzed by DNA methyltransferases using S-adenosylmethionine (SAM) as the methyl donor. DNA methylation plays a crucial role in various cellular processes such as gene imprinting, chromosome stability, and gene transcription (Javier, 2020). The fundamental mechanisms underlying the maintenance of DNA methylation in plants have been extensively reviewed elsewhere. In Arabidopsis thaliana, the preservation of the DNA methylation pattern involves all five DNA methyltransferases. In summary, CHG and CHH methylation are sustained through self-reinforcing loops that include a DNA methyltransferase (such as CMT3, CMT2, DRM2, or DRM1), histone methyltransferases, and nucleosomes marked by repressive modifications like H3K9me2 and H3K9me1 (as discussed in other reviews). Conversely, the maintenance of CG methylation predominantly depends on the DNA methyltransferase itself—specifically, MET1 in Arabidopsis thaliana (Muhammad et al., 2019; Zangi et al., 2020). It is proposed that MET1 acts on hemimethylated CG sites during DNA replication, catalyzing CG methylation on the nascent DNA strand. Post-translational covalent reactions, including methylation, acetylation, phosphorylation, ADP-ribosylation, ubiquitination, and sumoylation, occur at the N-terminal and C-terminal regions of histones H3 and H4, impacting chromatin structure and gene expression (Javier, 2020). Previous studies have revealed that DNA methylation readers, specifically SU(VAR)3-9 homologs SUVH1 and SUVH3, the SUVH proteins bind to methylated DNA and recruit the DNAJ proteins to enhance proximal gene expression, thereby counteracting the repressive effects of transposon insertion near genes. In specific conditions can enhance gene expression (Nie, 2021; Sun et al., 2022).

Heavy metal stress affects DNA structure, stability, and gene expression regulation. In plants, DNA methylation changes aid in adapting to heavy metal stress, particularly cadmium stress (Xin et al., 2019; Pan et al., 2024). Studies suggest that cadmium treatment increases DNA methylation levels in rice, Arabidopsis, kelp, and barley, enhancing plant tolerance to Cd (Lee et al., 2021). Several transport proteins involved in cadmium absorption, transport, compartmentalization, and detoxification in plants have been identified. Cadmium stress induces metal transport proteins and reactive oxygen species scavenging enzymes as the primary functional proteins (Kazemi et al., 2020). The accumulation and tolerance of heavy metals in plants are governed by a highly complex regulatory network involving numerous genes. Recent research on rice, Arabidopsis, and other plants has uncovered multi-layered transcriptional networks, including transcription factors (TFs), long non-coding RNAs (lncRNAs), and microRNAs (miRNAs), that respond to cadmium stress (Quinn and Chang, 2016; Wen et al., 2020).

Feng et al (2016) conducted a study utilizing high-throughput single-base resolution bisulfite sequencing (BS-Seq) and RNA-Seq to analyze the DNA methylation patterns in cadmium-treated rice seedlings. Differential methylation was observed in genes responsible for metal transport proteins, Cd detoxification proteins, and metal-related transcription factors, suggesting their involvement in regulating rice tolerance to cadmium stress. In a study by Sun et al. (2022), it was observed that grafting had a significant impact on reducing the accumulation of total sulfur and cadmium in soybean through the mediation of DNA methylation. The decrease in methyltransferase gene expression led to a decline in the expression of genes related to sulfur metabolism, with the S-adenosylmethionine (SAM) gene being particularly affected. These findings indicate the involvement of DNA methylation in the reduction of total sulfur and cadmium content (Javier et al., 2020).

### 3.2 The role of chromatin remodeling in plant cadmium stress

Chromatin, the repeating unit of nucleosomes, consists of a complex of DNA and proteins organized within the cell nucleus. It is formed by tightly condensed DNA wrapped around nuclear proteins known as histones. A nucleosome is defined as 146 base pairs of double-stranded DNA coiled around an octamer of histone proteins. The degree of chromatin condensation significantly influences the accessibility of transcription factors and DNA-binding proteins to DNA, thereby impacting their functional roles. Thus, chromatin remodeling refers to the rearrangement of chromatin from a condensed state to a transcriptionally accessible form, facilitating the access of transcription factors and other DNA-binding proteins to DNA and regulating gene expression (Niekerk et al., 2021). Chromatin remodeling is closely associated with the epigenetic modification of histone proteins through processes such as demethylation/methylation and acetylation/deacetylation, which can alter chromatin structure and subsequently activate or repress transcription (Ojolo et al., 2018). Understanding cadmium’s role during chromatin remodeling involves two significant pathways that regulate DNA replication and nucleosome stability within the chromatin structure. One pathway depends on the histone gene repressor (HIRA), while the other is mediated by chromatin assembly factor 1 (CAF-1); both pathways can be affected by Cd stress. The variability in the chemical properties and reactive toxicities of different metals suggests that a uniform mechanism of action for all toxic metals is unlikely (Zhang et al., 2012; Wu et al., 2022; Amogh et al., 2022). Heavy metals, including Cd, induce cytotoxic and genotoxic effects by disrupting the structures and functions of histones and other proteins, primarily by targeting thiol groups and inducing conformational changes (Nouairi et al., 2019). These alterations generally result in the inhibition of DNA replication, gene expression, and cell division. The heterotrimeric CAF-1 chaperone complex in Arabidopsis thaliana, for example, targets acetylated histone H3/H4 to nascent DNA strands for the *de novo* assembly of nucleosomes (Zhong et al., 2022). High levels of histone acetylation are often associated with transcriptionally active chromatin, while deacetylated histones are linked to inactive chromatin regions (Yang et al., 2018). In Arabidopsis, the heterotrimeric CAF-1 (Chromatin Assembly Factor 1) complex targets acetylated histones H3/H4 to newly synthesized DNA strands for *de novo* nucleosome assembly (Pitzschke et al., 2009). Shafiq et al. investigated the interplay between histone acetylation and DNA methylation, focusing on metal stress tolerance dynamics in Zea mays (Shafiq et al., 2020). Their research demonstrated that Zn, Cd, and Pb differentially regulated the expression of DNA methyltransferases and various histone deacetylases, ultimately suggesting caution in the excessive use of zinc fertilizers.

The diverse chemical properties and toxic responses of metals indicate the unlikelihood of a unified mechanism for all toxic metals (Sun et al., 2022). Heavy metals, including cadmium, disrupt the structures and functions of chromatin and proteins by attacking thiol groups in histones and other proteins. These conformational changes contribute to the cytotoxic and genotoxic effects of heavy metals, including cadmium (Niyikiza et al., 2020; Sun et al., 2022). The overall consequence of these conformational changes is the inhibition of DNA replication, gene expression, and cell division. Zabka et al., (2021) examined the epigenetic changes in transcriptional nucleosome assembly during the S-phase of the cell cycle by exposing apical root meristem tissues from fava bean seedlings to CdCl2. The results unveiled the interplay between cellular responses to cadmium toxicity, biochemical reactions, and the generation of reactive oxygen species (ROS) induced by DNA damage-related replication stress.

Various forms of chromatin modifications are possible during cadmium stress in plants, such as acetylation, methylation, phosphorylation, and ubiquitination. These modifications can alter the structure of chromatin and the accessibility of transcription factors, thereby influencing gene expression (Niyikiza et al., 2020). For example, high levels of histone acetylation are often associated with transcriptionally active chromatin, while deacetylated histones are linked to inactive chromatin regions (Yang et al., 2018). In Arabidopsis, the heterotrimeric CAF-1 (Chromatin Assembly Factor 1) complex targets acetylated histones H3/H4 to newly synthesized DNA strands for *de novo* nucleosome assembly (Pitzschke et al., 2009). Shafiq (2019) focused on investigating the dynamics of metal stress tolerance and the interplay between histone acetylation and DNA methylation. Their research revealed that Zn, Cd, and Pb can modulate the expression of different histone deacetylases, which, in turn, regulate the expression of DNA methyltransferases. This mechanism helps prevent excessive use of zinc fertilizers. In another study, Zabka et al. (2021) examined the epigenetic changes in transcriptional nucleosome assembly during the S-phase of the cell cycle by exposing apical root meristem tissues from fava bean seedlings to CdCl2. The results unveiled the interplay between cellular responses to cadmium toxicity, biochemical reactions, and the generation of reactive oxygen species (ROS) induced by DNA damage-related replication stress.

### 3.3 The influence of cadmium on histone acetylation

Histone acetylation is one of the first epigenetic mechanisms that have been extensively studied and found to be involved in transcriptional regulation (Verandra et al., 2021). It plays a role in diverse cellular processes, such as cell cycle progression, DNA repair, gene silencing, growth and development, flowering and seed development, and responses to biotic and abiotic stresses (e.g., salt, cold, and drought stress) (Huang et al., 2019; Verandra et al., 2021). Histone acetylation and deacetylation are dynamic and reversible processes. These processes are catalyzed by two classes of enzymes, namely histone acetyltransferases (HATs) and histone deacetylases (HDACs), which act on lysine residues in the tails of histones. In general, histone acetylation is linked to transcriptional activation, whereas histone deacetylation is associated with transcriptional repression (Nichols and Welder, 1981; Qadir et al., 2014; Shafiq et al., 2020; Zhang et al., 2024).

Emerging evidence indicates that plant HATs and HDACs have crucial functions in regulating gene expression during plant development and in response to environmental stresses (Luo et al., 2020). Moreover, studies have demonstrated that HATs and HDACs interact with multiple chromatin remodeling factors and transcription factors that contribute to the transcriptional regulation of various developmental processes. In a study by Lee (2017), the overexpression of *OsSNAT1* in transgenic rice was investigated. Conversely, Zabka et al., (2021) examined the potential correlation between cadmium (II)-induced oxidative stress and damage at the V chromosome genome level.

The bean seedlings underwent cadmium treatment and stress recovery in a hydroponic system. Exposure to cadmium toxicity resulted in two types of cadmium-induced secondary stress: oxidative stress, characterized by the critical role of reactive oxygen species (ROS) in genomic DNA disruption, ultimately leading to replication stress. Phosphorylation of histone H2AX at Ser-139 (*γ*-H2AX) is regarded as an early response in the cellular sensing/signaling pathway triggered by DNA double-strand breaks (Lee et al., 2017; Seneviratne et al., 2019; Zabka et al., 2021).

### 3.4 Regulation of miRNA and lncRNA during cadmium stress in plants

MicroRNAs (miRNAs) play a pivotal role in the modulation of plant growth and development, especially in the context of biotic and abiotic stressors (Sunkar et al., 2012; Zhao et al., 2015);. A collection of miRNAs has been characterized as differentially expressed miRNAs (DEMs) in response to cadmium (Cd) stress in different plant species (Zhou et al., 2020). For example, small RNA transcriptome profiling revealed that miR397, miR398, miR169 and miR9560 were up-regulated, whereas Cd repressed the expression of miR171, miR390 and miR395 in the roots of Brassica parachinensis (Liu et al., 2020; Yu et al., 2021; Shahid et al., 2023). These miRNAs are thought to be involved in the plant’s Cd stress response by modulating the expression of their target genes. Understanding the intricate regulatory networks involving these miRNAs may provide insights into the molecular basis of plant tolerance to Cd and guide the development of strategies to enhance crop resilience to heavy metal toxicity.

In order to respond appropriately to heavy metal stress by controlling the uptake, efflux, translocation and sequestration of heavy metal ions, plants have evolved complex, multi-layered regulatory mechanisms (Kramer et al., 2020). At the post-transcriptional level, heavy metal stress responses have been repeatedly demonstrated to be primarily in the form of microRNA (miRNA)-directed gene expression regulation, with the highly conserved miRNAs miR160, miR167, miR393, miR395, miR396, miR399 and miR408 identified in Arabidopsis, (Brassica napus), maize (Zea mays), red clover (Medicago truncatula), alfalfa (Medicago sativa), rice (Oryza sativa), radish (Raphanus sativus), soybean (Glycine max), sunflower (Helianthus annuus) and wheat (Triticum aestivum) to respond to Cd stress (Huang et al., 2009; Xu et al., 2013; Fu et al., 2014; Gu et al., 2018; Gao et al., 2019; Joseph et al., 2021). Furthermore, the identification of miRNAs specific to the auxin pathway (miR160, miR167 and miR393), in addition to the sulphur (S), phosphate (PO4) and Cu stress-responsive miRNAs, miR395, miR399 and miR408, suggests the complexity of a potentially ‘shared’ or ‘common’ miRNA-driven molecular response to Cd stress across a wide range of evolutionarily distant plant species.

Long non-coding RNAs (lncRNAs) of plants are shown actively involved in response to various biotic and abiotic stresses by mediating the gene regulatory networks (Zhu et al., 2023). lncRNAs are a type of non-protein-coding RNAs that are longer than 200 nucleotides and lack significant open reading frames (Ponting et al., 2009). Compared with mRNAs, lncRNAs are usually expressed at lower levels and have strong tissue- or cell-specific expression (Quinn and Chang, 2016; Miao et al., 2020; Su et al., 2022). With the recent application of high-throughput sequencing technology and novel bioinformatics tools, a large number of lncRNAs have been identified and characterized in wheat in relation to various abiotic stresses, including heat stress (Xin et al., 2011), cold stress (Lu et al., 2020), alkaline stress (Wei L. et al., 2022), and drought stress (Li et al., 2022). Most importantly, there is growing evidence that an increasing number of lncRNAs are involved in plant responses to heavy metal stress Using high-throughput sequencing, some of the lead (Pb)-responsive lncRNAs were identified and characterized in poplar by Chen et al. (2022). In Brassica napus, 301 differentially expressed lncRNAs were identified in response to Cd stress using deep RNA sequencing, and the expression of three lncRNAs (TCONS_00091906, TCONS_00097191 and TCONS_00033487) were significantly altered in Cd uptake and detoxification by qRT-PCR analysis (Feng et al., 2016). In addition, LncRNA28068.1 and LncRNA30505.2 from Betula platyphylla were likely to enhance Cd tolerance by controlling their respective target genes L-lactate dehydrogenase A (LDHA) and heat shock protein (HSP18.1) (Wen et al., 2020). Although many lncRNAs have been shown to play essential roles in plant response to heavy metal stress, the potential regulatory roles of lncRNAs in wheat response to Cd stress are still unknown. Therefore, further research on Cd tolerance-associated lncRNAs in wheat is needed to better elucidate the regulatory mechanisms of plant response to Cd stress.

## 4 Summary and outlook

Epigenetics contributes to phenotypic variation. Understanding the effects of epigenetics and epigenomics on the plant phenotype can provide insights into the influence of environmental factors (Liu et al., 2023). Extensive documentation and updates have been made regarding the understanding of plant tolerance to cadmium, encompassing the physiological and biochemical effects as well as responses to cadmium toxicity. The increased uptake of cadmium by plant cells predominantly leads to reductions in plant growth, development, and yield. Breeders can utilize existing epigenetic variations or modify the epigenome to address the ongoing problem of genetic erosion and discover concealed variations. Researchers have successfully identified key genes and proteins that regulate cadmium tolerance in various plants as a means to improve plant growth under high cadmium concentrations (Rutger and Marianne, 2020; Zheng et al., 2022).

In various crop species, the initial identification of natural epigenetic variants controlled important traits such as fruit ripening in tomatoes, vitamin E content, sex determination in melons, and dwarfism in rice, laying the foundation for the connection between epigenetics and crop improvement. Since then, natural epigenetic variation has been described in various crops and linked to different traits and environmental factors. For example, sweet cherry (Prunus avium) varieties exhibit a wide range of phenotypic variation in valuable traits such as fruit size, shape, and sugar content, with high epigenetic diversity found in wild populations. Notably, the epigenetic index has been found to correlate with more phenotypic parameters compared to the genetic index, suggesting that epigenetic variation may contribute more to phenotypic variation than genetic variation (Sarah et al., 2022).

**Figure 2:**
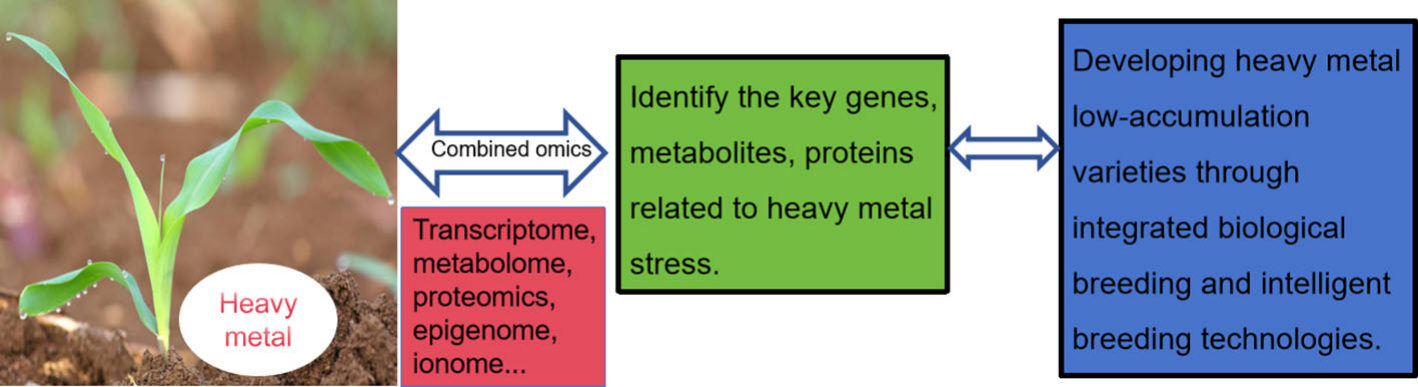
Developing plant varieties with low metal accumulation by utilizing multi-omics and biotechnology techniques.

Epibreeding programs have the capacity to generate significant phenotypic variations within a single generation. Some of these variations can be inherited from one generation to the next, ultimately overcoming any limitations that may hinder the effectiveness of crop breeding programs. Besides these molecular mechanisms, the significance of epigenetic regulation has come to light as a crucial and intricate factor in how plants respond to heavy metal stress. Hence, it is crucial to develop more robust bioinformatics pipelines for analyzing plant epigenetics under cadmium stress (Serena et al., 2022). Considering the current emphasis on genetic factors in breeding technologies, there is a potential for novel avenues in crop development by incorporating epigenetic information at the level of epiallelic variations. Nevertheless, to gain deeper insights into the mechanisms underlying the initiation and perpetuation of epigenetic diversity in crops, it is imperative to conduct extensive research across diverse plant species. Development of varieties that are more resilient to environmental stressors. Developing plant varieties with low metal accumulation is a critical goal in agriculture and environmental management, particularly in areas affected by heavy metal contamination. Using multi-omics approaches and biotechnology techniques, researchers are making progress in breeding plant varieties with the ability to accumulate low levels of heavy metals. Multi-omics techniques, including genomics, transcriptomics, proteomics and metabolomics, provide a comprehensive view of the complex molecular mechanisms underlying metal uptake, translocation and detoxification in plants (**Figure 2**). These approaches enable scientists to identify key genes and proteins involved in heavy metal tolerance and accumulation, and to understand how these elements affect plant metabolism and growth.

Whole genome sequencing, single cell epigenomics and epigenetic genome editing tool CRISPR off, which are emerging as key tools in contemporary research, offer advanced strategies for deciphering epigenetic regulation in plants under heavy metal stress (Nuñez et al., 2021; Babar et al., 2024; Martino et al., 2024). Whole genome sequencing allows a thorough examination of an organism’s genetic blueprint, enabling the identification of genes and regulatory elements that may play a role in a plant’s response to heavy metals (Růzicka et al., 2017; Guo et al., 2022). At the same time, single-cell epigenomics allows the study of epigenetic changes at the level of individual cells, providing a granular view of the different responses of plant cells to heavy metal stress. This includes the analysis of DNA methylation, histone modifications and non-coding RNA expression patterns, which show significant variation between cells and are essential for elucidating cellular stress response and adaptation mechanisms (Hu et al., 2022). By synergizing data from whole genome sequencing and single cell epigenomics, researchers will be able to identify specific genes and epigenetic signatures that correlate with heavy metal tolerance. This knowledge can be used to enhance a plant’s innate defenses against heavy metals through targeted genetic modification. For example, the introduction or modification of genes already involved in heavy metal tolerance, together with the use of genetic markers associated with such tolerance, can enhance the selection of plants with improved resistance in breeding programs. Such an approach not only strengthens plants’ natural defenses against heavy metals, but also paves the way for the development of varieties that are more resilient to environmental stressors.

Developing plant varieties with low metal accumulation is a critical goal in agriculture and environmental management, particularly in areas affected by heavy metal contamination. Using multi-omics approaches and biotechnology techniques, researchers are making progress in breeding plant varieties with the ability to accumulate low levels of heavy metals. Multi-omics techniques, including genomics, transcriptomics, proteomics and metabolomics, provide a comprehensive view of the complex molecular mechanisms underlying metal uptake, translocation and detoxification in plants (**Figure 2**). These approaches enable scientists to identify key genes and proteins involved in heavy metal tolerance and accumulation, and to understand how these elements affect plant metabolism and growth.
